# Where Have I Been? Where Should I Go? Spatial Working Memory on a Radial Arm Maze in a Rat Model of Depression

**DOI:** 10.1371/journal.pone.0062458

**Published:** 2013-04-22

**Authors:** Sophie Helene Richter, Benjamin Zeuch, Katja Lankisch, Peter Gass, Daniel Durstewitz, Barbara Vollmayr

**Affiliations:** 1 Research Group Animal Models in Psychiatry, Department of Psychiatry and Psychotherapy, Central Institute of Mental Health, Medical Faculty Mannheim, University of Heidelberg, Mannheim, Germany; 2 Bernstein Center for Computational Neuroscience, Department of Psychiatry and Psychotherapy Central Institute of Mental Health, Medical Faculty Mannheim, University of Heidelberg, Mannheim, Germany; Tulane University Medical School, United States of America

## Abstract

Disturbances in cognitive functioning are among the most debilitating problems experienced by patients with major depression. Investigations of these deficits in animals help to extend and refine our understanding of human emotional disorder, while at the same time providing valid tools to study higher executive functions in animals. We employ the “learned helplessness” genetic rat model of depression in studying working memory using an eight arm radial maze procedure with temporal delay. This so-called delayed spatial win-shift task consists of three phases, training, delay and test, requiring rats to hold information on-line across a retention interval and making choices based on this information in the test phase. According to a 2×2 factorial design, working memory performance of thirty-one congenitally helpless (cLH) and non-helpless (cNLH) rats was tested on eighteen trials, additionally imposing two different delay durations, 30 s and 15 min, respectively. While not observing a general cognitive deficit in cLH rats, the delay length greatly influenced maze performance. Notably, performance was most impaired in cLH rats tested with the shorter 30 s delay, suggesting a stress-related disruption of attentional processes in rats that are more sensitive to stress. Our study provides direct animal homologues of clinically important measures in human research, and contributes to the non-invasive assessment of cognitive deficits associated with depression.

## Introduction

Major depression is characterized by persistent sadness or low mood and loss of interests or pleasure as core symptoms [1]. Diagnostic criteria also include cognitive impairments, such as reduced ability to concentrate and indecisiveness, but cognitive dysfunction associated with major depression has not received much attention until the last decade. Accumulating evidence indicates cognitive disturbances in the following domains: Affective processing, memory, negative feedback, and executive control, e.g. working memory [2]–[5]. Cognitive dysfunctions considerably contribute to impairment resulting from depression and may even serve as endophenotypes guiding us to understand mechanisms underlying depression.

To study cognitive dysfunction, we employ a genetic animal model of depression, the “learned helplessness” model [6]. Derived from a cognitive theory of human depression [7] and based on the premise that the experience of uncontrollable stress causes a helpless state with depression-like symptoms, this model possesses a high translational potential for the investigation of a cognitive outcome. By selective breeding of helpless and non-helpless rats, two strains were established: Congenitally helpless rats (cLH) and rats resistant to helplessness (cNLH; [8]), exhibiting differences in pathophysiological, neurochemical, and behavioural parameters [9]–[13]. Moreover, in two recent studies applying cognitive bias procedures, the depressive-like phenotype of cLH rats was found to manifest in a negative response bias [14], [15], indicating impaired affective processing similar to depressed patients. In the present study, we therefore aimed to test the hypothesis whether the observed depression-like symptoms in these rats are accompanied by deficits in executive control, focussing on working memory and attention.

To these ends, we employed a well-established multiple-item working memory and decision making task, namely the *delayed spatial win-shift* (DSWS) task. The ability to find and retrieve food efficiently in a minimum amount of time is an essential survival strategy for rodents. Therefore, it is not surprising that several studies have reported a remarkable ability of rats to remember spatial locations [16]. Using radial arm maze procedures [17], rats quickly learn to visit each arm only once in a session to retrieve a food reward. Accurate performance is thereby dependent on both memory for previously visited arms and a natural tendency not to revisit arms within a trial, referred to as “win-shift” strategy [18]. For the study of working memory, the original radial arm maze task has been extended to include a temporal delay imposed somewhere within the sequence of arm visits. Thus, the animals have to retain and retrieve information about (to be) visited arms not only on-line during actual arm selection, but also across a retention interval. Besides being well characterized behaviourally [19]–[21], the task has been extensively studied with regards to its neuroanatomical and neurophysiological basis [22]–[25]. Briefly, the test consists of three phases, training, delay and test, corresponding loosely to the three stages of working memory (encoding, retention, and retrieval). During the first phase (training), four out of eight arms are randomly blocked, and the remaining four arms are baited. Once a rat has retrieved the four rewards, it is put in the centre of the maze for a delay. In the following test phase all eight arms are open and the rat has to remember which arms were previously blocked and enter them to receive a food reward. Thus, the animal has to “win-shift” to solve the task correctly [18], [26]. Under such conditions choice accuracy is close to 100% over the first four choices, but decreases for choices five to eight presumably due to retrieval impairments [17], [27]. A crucial parameter of this task is the length of the temporal delay: Generally task performance decreases with the delay length, but shorter versus longer delays may also differentially evoke prefrontal cortex-dependent working memory versus hippocampus-dependent intermediate memory [19], [21], [28]–[31]. To compare working memory capabilities in our helpless and non-helpless rats, we have therefore adopted a DSWS procedure with delay in the middle of the choice sequence, and have introduced two retention intervals, 30 seconds and 15 minutes, respectively.

## Materials and Methods

### Subjects

We used 31 males of congenitally helpless (cLH, n = 15) and non-helpless (cNLH, n = 16) rats from different litters of the 72^nd^, 73^rd^ and 74^th^ generations of the colonies bred at the Central Institute of Mental Health in Mannheim. Originally, both strains were bred from Sprague-Dawley rats by selecting animals susceptible to helplessness and animals resistant to the effects of uncontrollable stress. All rats had been tested for learned helplessness at the age of nine weeks to confirm the helpless or non-helpless phenotype in an escape paradigm. The testing procedure as well as the origin and selective breeding of these strains have been described in detail elsewhere [8], [32]. Briefly, the test (in boxes from TSE, Bad Soden, Germany) consisted of 15 trials in which an electric foot shock (0.8 mA, 60 s) could be terminated by the animals pressing a bar. Trials not stopped after 20 s were considered as a failure. Animals with nine or more failures were considered to be helpless, while animals with less than five failures were considered to be non-helpless. cNLH rats were exposed to a series of randomized, unpredictable and uncontrollable 0.8 mA shocks summing up to a total of 20 min 24 h prior to the test for learned helplessness.

Rats were housed in groups of two in conventional standard macrolon cages (Type IV) with sawdust (Rehofix MK-2000, JRS J. Rettenmaier & Söhne GmbH+Co. KG, Rosenberg, Germany), additional nesting material in form of two soft tissue papers, standard rat diet (Ssniff R/M-H, Ssniff Spezialdiäten GmbH, Soest, Germany) and tap water *ad libitum*. The colony room was maintained at a temperature of 22±1°C, a relative humidity of 50±5%, and a 12 h light-dark cycle with the lights off at 19∶00.

To test our predictions in a wide range of rats and to avoid unnecessary breeding, we used a heterogeneous group of rats of different generations varying in age (10–16 weeks of age at the start of testing), ad libitum feeding body weight (350–620 g), and housing experience prior to testing (different housing densities) (see [33]–[35]) To account for this variation in our experimental design, rats were allocated to same-strain cages of two according to these variables. Thus, within a cage rats were as homogeneous as possible, while rats of different cages varied with respect to these variables. According to a split-plot-concept, cagemates were randomly allocated to either the 30-s- or 15-min-retention interval. Furthermore, ‘cage nested within strain’ was included in the statistical analysis to eliminate between-cage variation that would otherwise be assigned to error.

Depending on the position in the rack, cages may differ in local environmental conditions (e.g. temperature, humidity, lighting, and disturbance) due to variation in proximity to ventilation, lights and human traffic. To avoid position bias, we controlled for cage position in the experimental design [36]. Thus, the cages were stacked in horizontal lines of three cages in one rack, with cages of cLH and cNLH rats balanced for horizontal and vertical position in the rack.

Prior to testing, *ad libitum* feeding weights were obtained and rats were then food restricted to 85% of these initial weights during testing. To maintain the animals in a healthy state and to adjust the daily amount of food individually, weight and health status of each animal were checked on a daily basis. However, since most of the rats were initially overweight, food restriction did not cause any observable changes in the animals’ behaviour.

### Ethical Statement

All procedures complied with the regulations covering animal experimentation within the EU (European Communities Council Directive 86/609/EEC). They were conducted in accordance with the institutions' animal care and use guidelines and approved by the national and local authorities (Regierungspräsidium Karlsruhe, permit number: 35-9185.81/G-204/11). Moreover, all efforts were made to minimize the number of animals used and the severity of procedures applied in this study.

### Experimental Design

cLH and cNLH rats were tested daily on 20 consecutive days, including two days of habituation followed by 18 days of testing on the DSWS task. Since no more than eight rats could be tested per day, rats were tested in four consecutive replicates (n = 4/strain). Moreover, to contrast two different delay lengths, half of the animals were tested with a 30 s delay, while the other half was locked in the centre of the maze for a delay length of 15 min. The delay length was balanced between individuals and strains with the two rats per cage always being trained to different delays. In a 2 × 2 factorial parallel design, we therefore investigated the DSWST performance of rats belonging to four independent experimental groups: cNLH 30 s (n = 8), cNLH 15 min (n = 8), cLH 30 s (n = 7), cLH 15 min (n = 8).

### Delayed Spatial Win-Shift Test

#### Apparatus

We used an eight arm radial maze (arm length 50 cm, arm width 14 cm, hub diameter 40 and height 40 cm, raised 50 cm above the floor) made of dark grey Perspex with manually operating guillotine doors separating the central area from each of the eight arms. The maze was placed in a different testing room close to the colony room, where stable dim light (30 l×) could be provided and behavioural observations could be done without any external disturbances. A screen was used to divide the testing room in two areas, mainly to reduce disturbances due the experimenters’ presence in the testing room. The guillotine doors could be opened/closed using a pulley system operated from behind this screen so that the experimenter was not visible to the rats during training and testing. The behaviour was recorded using an overhead camera mounted above the maze and linked to a monitor behind the screen, allowing the experimenter to perform either behavioural live observations or later video-based analyses. Because previous experiments had shown that rodents rely mainly on extra-maze cues to identify arms [21], [22], a variety of black and white geometric shapes were located on the walls of the testing room.

At the beginning of each trial, the appropriate guillotine door was opened to a height of 15 cm and the rat was allowed to enter the arm. Each arm was partly confined by a tunnel-like plastic cover (cover length 20 cm, cover height 20 cm) to prevent the rats from climbing from one open arm to the other instead of entering a new arm out of the centre without reducing the visual perception of the apparatus as a whole. Furthermore, these enclosed parts reduced the aversive character of the arms facilitating the habituation to the apparatus in the first phase of the experiment. Located 4 cm from the end of each arm, there were recessed goal pots (diameter 4 cm, depth 4 cm), which could be removed and cleaned between the phases.

### Procedure

We based our protocol on that previously described by Seamans and colleagues [22].

#### Habituation

Before habituating the rats to the apparatus, we familiarized them with the fruitloops used as reward in the task (Fruit Rings, Crownfield, Nordgetreide GmbH & Co. KG, Lübeck, Germany). This was done by placing six fruitloops in each cage of two rats for three consecutive days. Rats were then pre-exposed to the apparatus on two consecutive days in order to ensure familiarity with the apparatus, with being enclosed in the centre of the maze, and with obtaining fruitloops on the maze. At the beginning of each habituation session, an individual rat was placed in the centre of the maze. After one minute, all eight doors were opened simultaneously and the rat was allowed to explore the whole apparatus for a maximum of ten minutes. All eight goal pots were baited and according to the classical radial arm maze procedure [37], rats were allowed to move freely and obtain the food rewards. The habituation phase was completed when either all eight fruitloops had been retrieved or ten minutes had elapsed. We recorded the number of arms visited (defined as a rat placing all four paws on the arm), the number of faecal boli dropped, and the number of fruitloops retrieved (max. 8).

#### Testing

**Figure 1:**
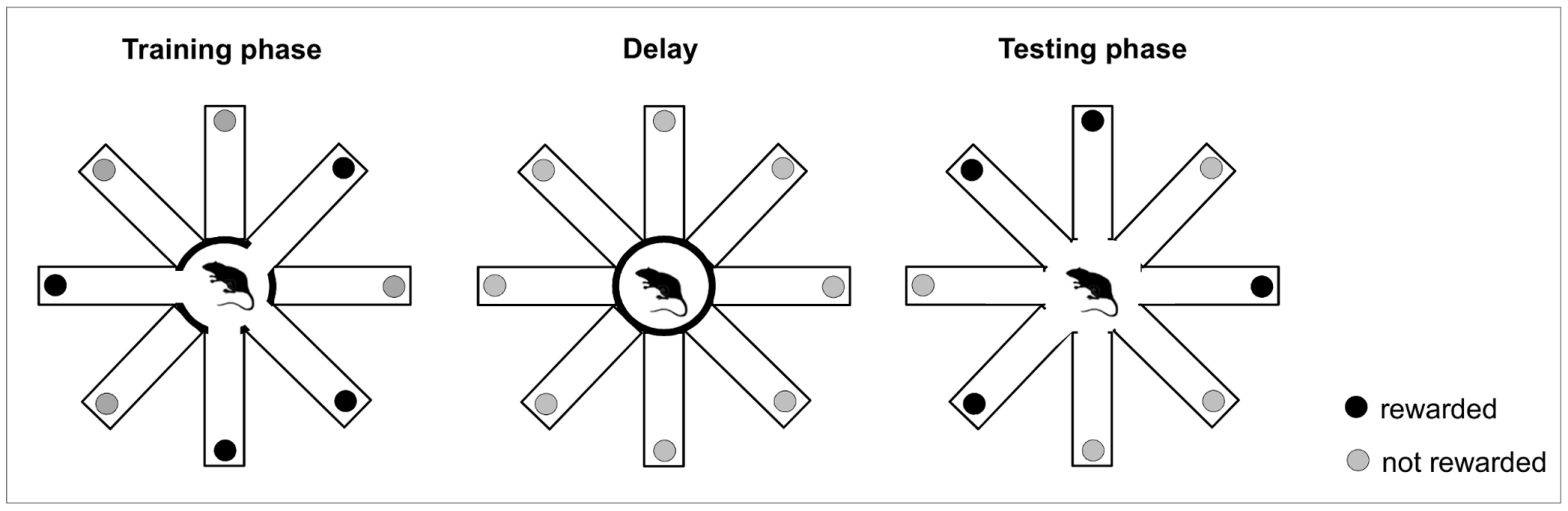
DSWS testing procedure. The DSWS test consisted of a training and a test phase separated by a delay that was either 30 seconds or 15 minutes long.

Subjects were tested once per day on 18 successive days. Each trial consisted of three phases: Training phase, delay, and test phase, always conducted in the same way (**Figure 1**). In the training phase, four randomly selected arms were baited, while the other four arms were blocked by the guillotine doors. The rats were allowed to enter the four open arms and retrieve the fruitloop in a period of no more than five minutes. Upon retrieving all four rewards, the animal was locked in the centre of the maze for a delay period of either 30 seconds or 15 minutes according to the experimental group (see experimental design). After the delay, all eight doors were opened simultaneously and the animal began the test phase. In this phase the fruitloops were placed on those arms that had been blocked during the training phase (**Figure 1**). The rat was thus expected to enter the arms that had not been visited in the training phase ( = win-shift strategy). The test phase started with opening the doors, allowing the rat to enter the arms, and ended with the rat retrieving the last fruitloop or reaching a cut-off point of 300 s.

Testing order was randomized on a daily basis and in order to avoid any possible experimenter bias, the whole experiment was run as a blind experiment. Altogether, four experimenters conducted the experiments, each being adept in working with rats and conducting behavioural tests.

At the beginning of each test session all rats were transported to the test room in their home cages and allowed to acclimatize to the room for 15 min before testing commenced. Testing was done during the light phase of the cycle and the test session was finished with the last animal being tested on the maze and put back into its home cage. Before each trial the whole apparatus was cleaned with alcohol (antifect ® N liquid, Schülke & Mayr GmbH, Norderstedt, Germany). Once a rat had completed a trial, it was put back in the centre before removing it from the maze and preparing the apparatus for the next animal.

Performance of the subjects was noted on standard sheets recording two time measures, the order of arm choices and the retrieval of fruitloops. From this, the following behavioural measures were analysed for both the training and the test phase: time to complete a phase (s), latency to retrieve the first fruitloop (s), number of arm entries (a subject places all four paws on the respective arm), number of correct arm entries (arm entry with fruitloop retrieval), and errors made (any incorrect arm entry). Errors were further classified as follows:

Within-phase error (WE): Re-entry of an arm that was baited within the same phase, reward was retrieved at first entry

Across-phase error (AE): Re-entry of an arm that was baited during the training phase, reward was retrieved in the training phase (max. 4)

In order to correct error counts for the total number of arm entries, an overall memory score was calculated as follows:
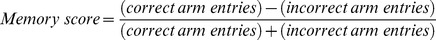



On a scale from −1 to 1 this score describes individual memory performance with a score of 1 reflecting perfect performance and a score of −1 indicating the opposite ( = all arm entries incorrect). We further calculated relative error scores (number of errors/number of arm entries) to compare errors of different types (WE, AE) and phases (training, test) among each other.

As individual learning curves are usually monotonically increasing, starting from a low baseline, progressing through an accelerating learning phase, and then levelling off at some plateau, they can usually be well described by sigmoid-type functions [38]. Therefore, sigmoid functions of the form
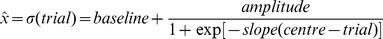
were fit through a least-squared-error criterion (with constraints on the minima and maxima of parameters derived from the data) using purpose-written Matlab routines to the time series (*x*) of observed performance scores. The four free parameters (regressors) ‘baseline’, ‘amplitude’, ‘centre’, and ‘slope’ determined by the fitting refer to the minimum of the sigmoid curve (baseline performance), maximum minus minimum of the curve (total learning improvement), the trial (day) where learning has halfway been achieved and learning progress is steepest, and the slope by which the sigmoid depends on the trial number ‘trial’. Additionally, the curve maximum ( = baseline+amplitude) was considered in some analyses. These curves thus neatly summarize, in a statistically robust manner, individual learning performance by just four parameters. For characterizing learning improvement, however, the actual learning amplitude, that is the actual difference between the maximum and the minimum of the curve *across the 18-trial training window*, was used (instead of the theoretical amplitude of the sigmoid curve that one would get across a trial-range from minus to plus infinity).

### Data Analysis

All data were analysed using General Linear Models (GLMs). To meet the assumptions of parametric analysis, residuals were examined graphically for homoscedasticity and outliers, and using the Kolmogorov-Smirnov and Shapiro-Wilk tests for normal distribution. When necessary, the raw data were transformed using logarithmic transformations. Analyses were blocked by ‘cage nested within strain’ to control for cage effects and Type III SS are used throughout to ensure that all results generalize across the other factors in the model [39].

Specifically, for the habituation phase we averaged data over the two days for each rat and assessed the effects of strain on the outcome measures by using a GLM with ‘strain’ as fixed and ‘cage nested within strain’ as blocking factor.

To compare the amount of errors made during the two phases of the DSWST, we averaged relative error scores (errors/choices) for both the training and the test phase and calculated a repeated measures ANOVA with ‘error phase’ (2 factor levels: training, testing) as within-subjects factor, ‘strain’ (2 factor levels: cLH, cNLH) and ‘delay’ (2 factor levels: 30 s, 15 min) as between-subjects factor, and ‘cage nested within strain’ as blocking factor. Furthermore, errors made during the test phase can be either of within- or across-task-phase type, i.e. re-entering arms from the test or the previous training phase. We therefore averaged the relative numbers of within- vs. across-phase errors over the eighteen trials and calculated a repeated measures ANOVA with ‘error type’ (2 factor levels: within-phase error, across-phase error) as within-subjects factor, and ‘delay’, ‘strain’ and ‘cage nested within strain’ as between-subjects factor.

Memory performance as a function of time (learning) and time measures were also analysed using repeated measures ANOVAs. To yield statistically more robust performance measures as a function of time, temporal averaging was applied with blocks of three consecutive days combined into one mean value. Pre-analyses, however, have shown that effects are stable with respect to the specific chunking of trials. Besides ‘time’ (6 factor levels: Ø days 1–3, Ø days 4–6, Ø days 7–9, Ø days 10–12, Ø days 13–15, days Ø 16–18) as within-subjects factor, we included ‘strain’, ‘delay’ and ‘strain nested within cage’ as between-subjects factors. Behavioural measures were analysed summarizing data of both the training and the test phase or focusing on the test phase only. All statistical tests were conducted using the software package SPSS/PASW (version 20.0 for Windows) and differences were considered to be significant at *P*≤0.05.

## Results

### Habituation

Congenitally helpless and non-helpless rats did not differ with respect to the number of arms visited (F_1,15_ = 1.55, p>0.10) and the number of faecal boli dropped (F_1,15_ = 0.40, p>0.10). However, cLH rats were found to retrieve more fruitloops than cNLH rats (F_1,15_ = 6.99, p = 0.02), although overall numbers were rather low in both strains (**Table 1**).

**Table 1:** Measures assessed during the habituation phase.

	Fruitloopsretrieved [#]	Armsvisited [#]	Bolidropped [#]
**cNLH**	1.938±0.454	24.344±1.241	1.406±0.372
**cLH**	3.687±0.482	22.094±1.317	1.063±0.3

We recorded the average number of fruitloops retrieved, arms visited and faecal boli dropped of cNLH and cLH rats during the habituation phase of the delayed spatial win-shift test. Data are presented as means ± standard error of the means.

### Testing

#### Relative training and testing errors

We expected rats to make more errors in the test phase than in the training phase, both because there are more opportunities for making errors in the test phase due to the larger number of already exploited arms, and because of potential working memory decline during the delay. Indeed, performance was found to be significantly better in the training than in the test phase as indicated by a higher error score in the test phase (F_1,13_ = 107.33, p<0.001). Furthermore, we observed a delay-dependent effect on error scores with 15-min-animals making less errors than 30-s-animals (F_1,13_ = 5.97, p = 0.03). This was mainly due to deficits in the test phase (delay-by-error-phase-interaction, F_1,13_ = 7.26, p = 0.02). ‘Delay’ was also found to interact with ‘strain’ such that the overall error score was highest in cLH rats tested with a 30 s delay and lowest in cLH rats with a 15 min delay, while there was no difference between cNLH rats tested with 15 min and 30 s delays (delay-by-strain-interaction, F_1,13_ = 6.32, p = 0.03, Bonferroni post-hoc tests: cLH rats: p = 0.01, cNLH rats: p>0.10).

#### Temporal measures

**Figure 2:**
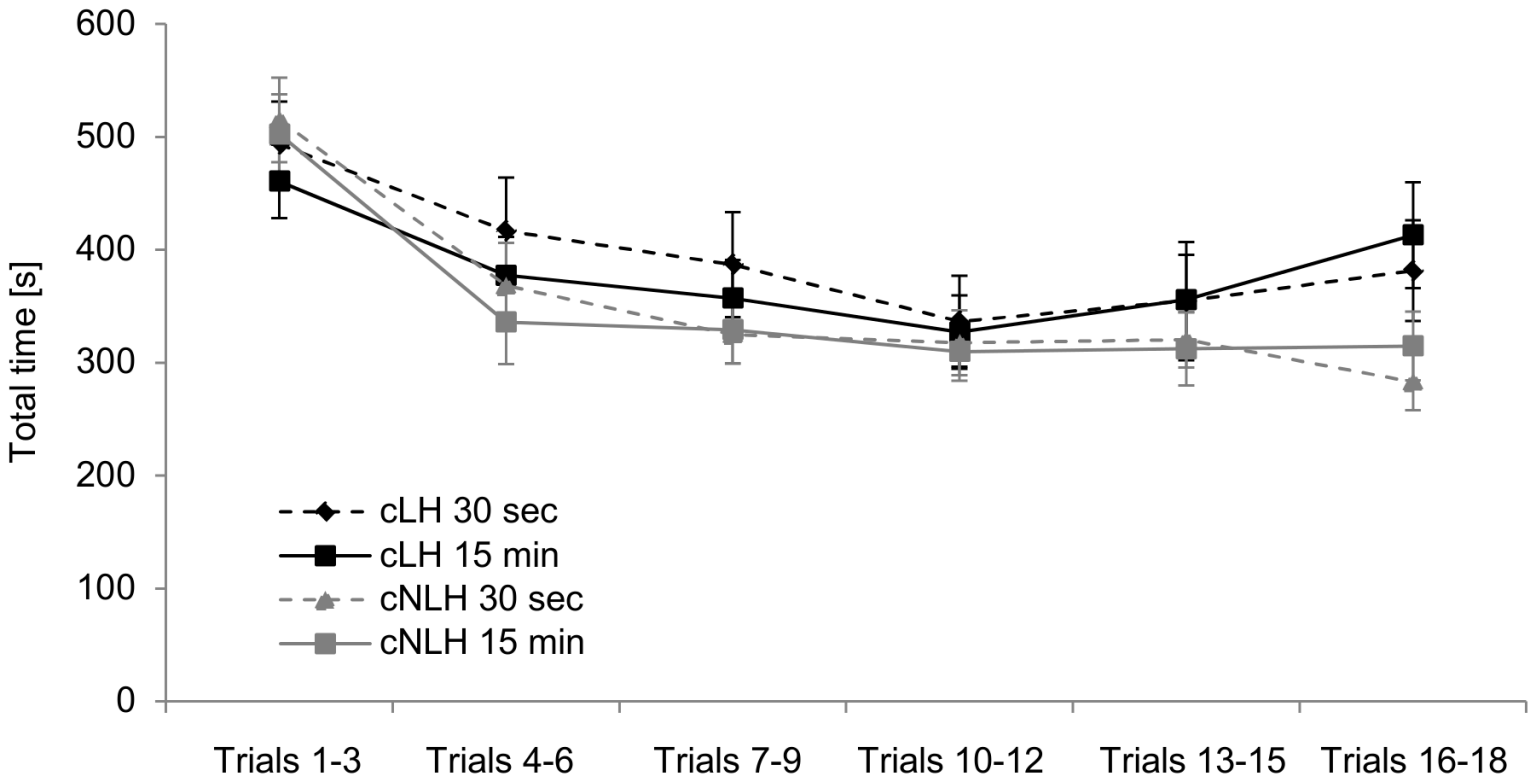
Total test time. The total time needed to complete a trial included both the training and the test phase. Data are averaged across three consecutive trials and presented separately for the four experimental groups as means ± standard error of the mean.

The total time needed to pass a complete trial including both training and test phase significantly decreased over time (F_5,65_ = 55.57, p<0.001). Furthermore, cLH required more time to go through a trial than cNLH rats (F_1,13_ = 5.85, p = 0.03), an effect that was mainly due to an increase of total test time in these animals over the last test days (time-by-strain-interaction, F_5,65_ = 7.73, p<0.001, **Figure 2**). Total test time was not affected by ’delay’ and we did not find any further interactions between ‘strain’, ‘delay’, and ‘time’ (p>0.10).

A separate analysis of the test phase, however, detected differential influences of ‘strain’ and ‘delay’. Thus, the time to complete the test phase was affected by both, ‘strain’ (strain: F_1,13_ = 4.89, p = 0.05; strain-by-time-interaction: F_5,65_ = 4.84, p = 0.001) and ‘delay’ (delay: F_1,13_ = 8.04, p = 0.01; delay-by-time-interaction: F_5,65_ = 1.95, p<0.10) with 30-s-rats requiring more time than animals tested with a retention interval of 15 min. However, because strains did not differ with respect to the number of arm entries made during the test phase (p>0.10), a general activity difference between cLH and cNLH rats seemed to be unlikely. By contrast, 30 s animals entered more arms in the test phase than animals tested with a 15 min delay (F_1,13_ = 5.80, p = 0.03), suggesting that the time difference could be explained by a higher number of arm visits in 30-s-animals.

We also observed delay-dependent (F_1,13_ = 4.41, p<0.10), but not strain-specific effects (p>0.10) on the latency to retrieve the first reward in the test phase. While rats tested with a 15 min delay took on average 29 s to retrieve the first fruitloop after opening the eight doors, the 30-s-animals required 48 s to retrieve a reward in the test phase. Looking at the variation across time revealed a general reduction of the latency across the 18 test days (F_5,65_ = 25.70, p<0.001), but also a significant interaction between ‘delay’ and ‘time’ (F_5,65_ = 2.96, p = 0.02) with 30-s animals displaying deficits particularly during the first week of testing. Although a similar stable performance was then reached in rats of both the 30 s and the 15 min condition, acclimatization to the procedure seemed to be clearly delayed in the 30 s group.

#### Working memory performance

**Figure 3:**
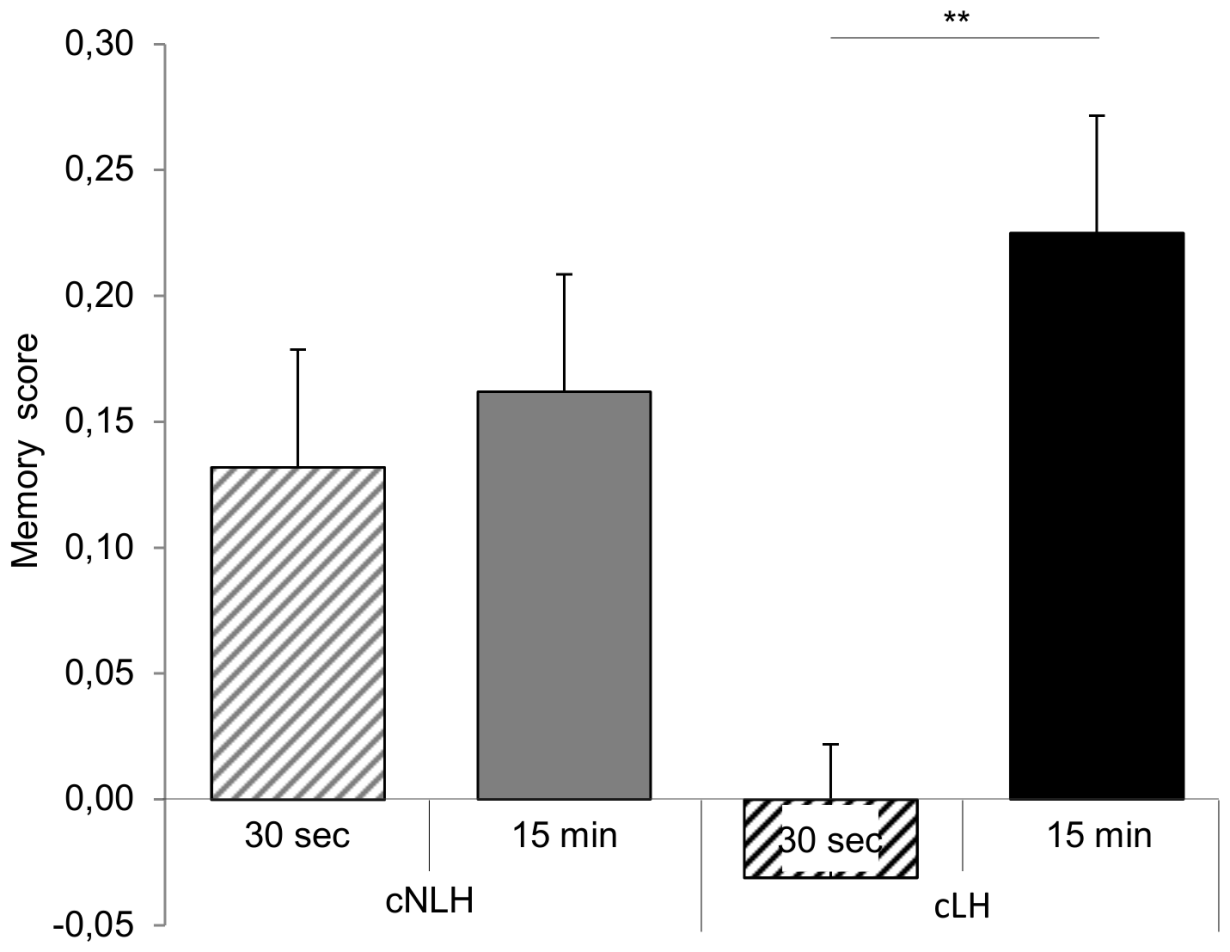
Overall memory score. The overall memory score was calculated on the basis of both the training and the test phase. Data are presented separately for the four experimental groups as means ± standard error of the mean, **p<0.01.

Working memory abilities were analysed using a memory score taking into account both correct (‘hits’) and incorrect (‘false alarms’) arm entries (for details see Materials & Methods). In the first three test days, rats started with an average memory score of −0.41, but consistently and significantly improved their performance on the maze until reaching an average score of 0.38 at the end of the testing including both the training and the test phase in the analysis (F_5,65_ = 87.81, p<0.001). Moreover, learning improvement was affected by ‘strain’ in that cNLH rats tended to learn the test principle faster, and to a reach a better memory performance than cLH rats at the end of the test phase (time-by-strain-interaction: F_5,65_ = 2.11, p<0.10). An overall main effect of ‘strain’, however, was not observed (p>0.10). Interestingly, we also found an effect of ‘delay’ on the memory score with animals of the 15 min delay performing better than animals of the 30 s condition (F_1,13_ = 8.74, p = 0.01). Further graphical and statistical examination revealed that this effect was particularly evident in cLH, while there was no difference between 30-s- and 15-min-animals of the cNLH strain (strain-by-delay-interaction: F_1,13_ = 5.45, p = 0.04, Bonferroni post-hoc tests: cLH rats: p = 0.003, cNLH rats: p>0.10, **Figure 3**).

**Figure 4:**
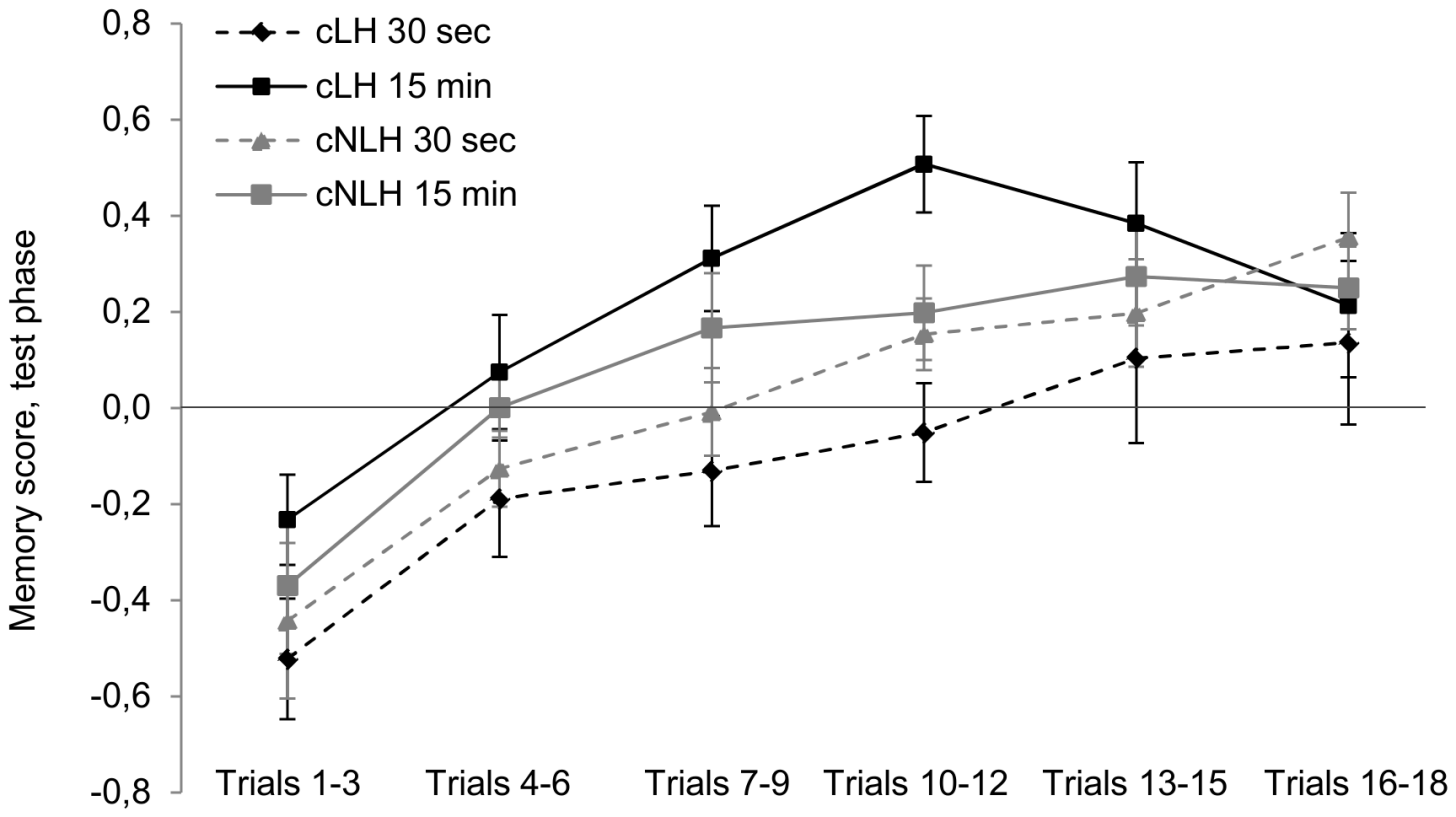
Memory score. The memory score was based on correct and incorrect arm entries during the test phase only. Data are presented separately for the four experimental groups as means ± standard error of the mean.

Focusing on memory performance in the test phase confirmed the interaction between ‘strain’ and ‘delay’ (F_1,13_ = 9.54, p = 0.01), showing that rats of the 30-s-condition have more difficulties than 15-min-rats with identifying the correct arms, an effect that was clearly more prominent in cLH than in cNLH rats (**Figure 4**). Furthermore, the repeated measures ANOVA revealed a main effect of ‘delay’ (F_1,13_ = 20.93, p = 0.001) as well as an interaction between ‘delay’ and ‘time’ (F_5,65_ = 2.14, p<0.10, **Figure 4**).

#### Error types

As anticipated from both the differences in *a priori* probabilities, and from the effect of the delay phase, rats were found to make more across-phase than within-phase errors during the test phase (F_1,13_ = 45.24, p<0.001), while we did not observe any effects of ‘strain’ or ‘delay’ on type of error (p>0.10).

#### Behavioural measures derived from sigmoid functions

**Figure 5:**
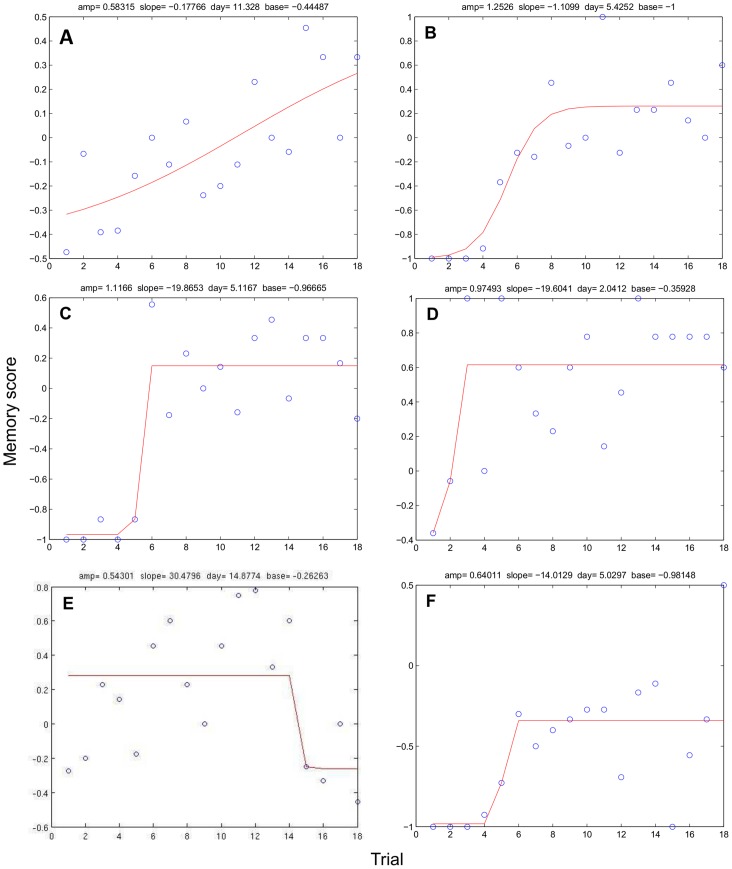
Sigmoidal curves. The examples of sigmoidal curves are based on the rats’ individual memory performance. Different types of learning curves were observed, ranging from gradual (**A, B**) to steep learning curves (**C, D**). Two exceptional cases were observed with one rat falling off in memory performance between trial 14 and trial 15 (**E**), and another one leveling off at a negative plateau value of −0.34 (**F**).

Finally, memory performance across the learning process (i.e., across days) was analysed by fitting sigmoid functions to the time series of daily total memory scores (i.e., including both the training and the test phase). A variety of different individual learning curves were observed, ranging from gradual to very steep ones (**Figure 5**). While some subjects followed a very smooth gradual learning process (**Figure 5A**), most learning curves were characterized by very fast transitions from chance to optimal performance within just a few days (**Figure 5B–D**). Baseline levels ranged from −1 to −0.04, and from there increased with an average amplitude of 0.86. In 50% of the subjects the curve had the steepest increase within the first five trials. Furthermore, while some animals reached a maximal learning score of 0.6, others reached an individual plateau already at about 0.1. Notably, one cLH rat of the 30 s condition did not exceed a plateau level of −0.34 (**Figure 5F**), while in one other case performance seemed to drop across trials as indicated by a negative slope of the fitted learning curve (**Figure 5E**).

**Figure 6:**
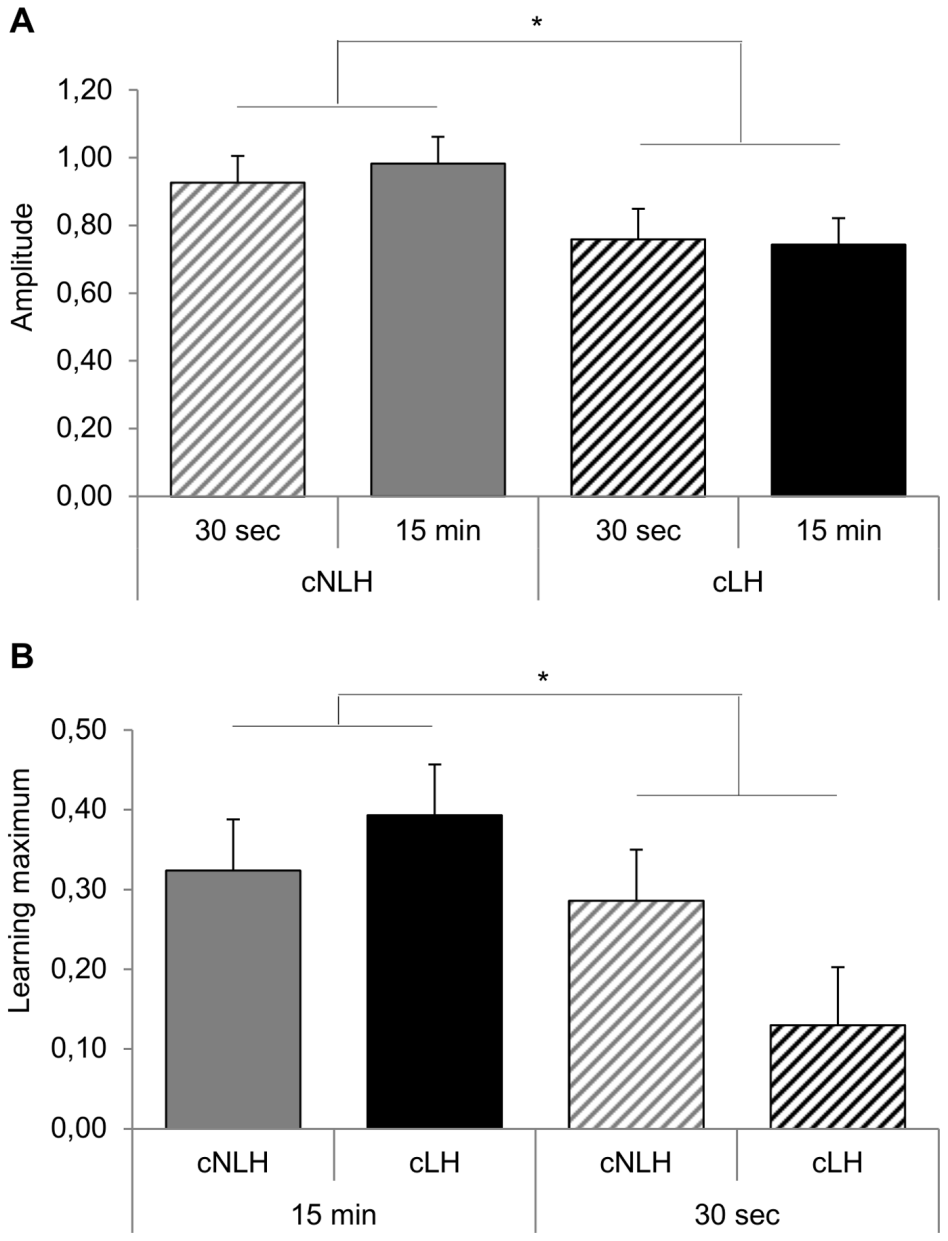
Learning measures. (**A**) The amplitude and (**B**) learning maximum were calculated on the basis of individual learning curves. Data are presented separately for the four experimental groups as means ± standard error of the mean, *p<0.05.

A GLM was calculated with ‘strain’ and ‘delay’ as fixed factors, and ‘cage nested within strain’ as blocking factor. Slope values were log-transformed to meet the assumptions of parametric analysis. We observed a significant main effect of ‘strain’ on learning *amplitude* (i.e. total learning improvement, F_1,13_ = 6.27, p = 0.03) with cNLH rats being characterized by a larger amplitude than cLH rats (**Figure 6A**). Furthermore, the maximal memory score significantly differed between the 15-min- and 30-s-conditions with animals of the 15 min condition reaching a higher plateau than rats of the 30 s delay (F_1,13_ = 5.17, p = 0.04, **Figure 6B**). However, we did not find any further effects of ‘strain’ and/or ‘delay’ on the remaining measures *centre* (i.e. the trial where learning has halfway been achieved and learning progress is steepest*)*, *baseline* (i.e. minimum of the sigmoid curve), and *slope* (i.e. learning speed, p>0.10).

## Discussion

The present study investigated working memory in a genetic rat model of depression, the “congenital learned helplessness” model [8], to shed light on cognitive aspects of information processing in a state of affective dysfunction. We employed an eight arm radial arm maze procedure with temporal delay of either 30 s or 15 min in the middle of the choice sequence.

### The DSWS test – a Tool to Study Higher Executive Functions in Rodents

As anticipated, more relative errors were made during the test phase than during the training phase. Moreover, errors made in the test phase were primarily caused by retrieval impairments as indicated by a higher ratio of across-phase errors in comparison to within-phase errors, confirming earlier studies (e.g. [40], [41]). Nonetheless, our findings reveal a basic ability of both strains to use previously acquired information after a temporal delay of either 30 s or 15 min. Thus, although the task performance was differentially affected by genotype and/or delay length, it steadily improved across the 18 trials in all groups, and reflected an increasing understanding of the test principle and/or the task procedure. Win-shift procedures therefore seem to capitalize on rats’ natural foraging strategies under conditions when food supply is dispersed [16], [18], [42], [43]. In light of research in animal neuropsychology, the test may offer the opportunity to assess core deficits associated with psychiatric disorders non-invasively and in close analogy to the human condition.

### Cognitive Deficits in a Rat Model of Depression?

In light of preclinical and clinical evidence for complex interactions between the cognitive and emotional domains [44], a methodologically sound approach to the assessment of executive functions requires the exclusion of affective state-associated differences in activity. Previous studies using this rat model had noted initial hyperactivity of cLH animals in the open field [9], and observed differences in running speeds between cLH and cNLH rats [15]. In line with these studies, we observed differences in the total test time with cLH rats requiring more time to complete a trial than cNLH rats. However, since cNLH and cLH rats did not differ in their arm choice activity, this is unlikely to interfere with the assessment of cognitive abilities. The fact that cLH rats on average needed more time per arm entry may rather be explained by diagnostic criteria, i.e. resistance to habituation, increased anxiety or higher levels of stress or arousal (e.g. [45]).

There has been a growing awareness that mood disorders are associated with distinct patterns of cognitive impairment [2]–[5], prompting the search for cognitive deficits in rats bred for learned helplessness. Indeed, cNLH rats tended to learn the test principle faster and to reach a higher memory score than cLH rats, further confirmed by a significant strain effect on the learning amplitude. However, strains did not differ with respect to the overall memory score, putting doubt on the presence of a systematic and stable difference in memory performance. This is in line with previous findings, showing that cLH rats also exhibit normal memory acquisition and retrieval in the Morris Water Maze [9]. Together, this argues against a general learning deficit in rats congenitally exhibiting the depressive-like phenotype. Furthermore, since accurate maze performance crucially relies on visual rather than on olfactory or self-movement cues [17], [46], this implies that there is no visual impairment in cLH rats.

Nonetheless, we observed an interaction between strain and delay length with respect to the overall memory score. While cLH rats tested with a retention length of 15 min showed a similar memory performance as cNLH rats of this condition, performance was clearly impaired in cLH rats after a short retention interval of only 30 s. Furthermore, we observed several strain-independent effects of the delay on the rats’ maze behaviour. Rats in the 30 s condition required more time to complete the test phase, and retention was clearly worse in these rats as indicated by lower memory scores and learning maxima. Moreover, rats tested with a short retention interval required more time to retrieve the first reward than rats tested with a 15 min delay. As these findings are in contrast to earlier findings revealing delay-dependent impairments in maze performance with increasing delay-duration [47], the involvement of a further factor, disrupting performance specifically in our short delay groups, seems to be likely.

Good maze performance in the long-delay groups argue for intact memory acquisition and recall abilities in both strains, pointing at attention deficits rather than working memory impairments in the short delay groups. Such attention deficits, however, may occur when subjects have to deal with several external challenges simultaneously. Although we did not explicitly apply a stressor, the use of the guillotine doors was observed to induce a brief freezing response, a behaviour typically shown by rats in stressful or frightening situations. The procedure of opening and closing the doors of the apparatus at the end of the training phase and the beginning of the test phase in combination with the cleaning in between may thus have constituted an uncontrolled intrinsic stressor that disturbed attention. In the 15 min condition these stressors were dispersed across time, allowing the animals to recover from the stressful stimuli in between. By contrast, the potentially stressful stimuli succeeded within a short time frame in the 30 s condition, and rats might not be able to recover completely from the first stressor before the last stressor occurred. As a consequence, attention was disrupted only in the short delay groups and maze performance was impaired after 30 s, but not after 15 min. Similarly, impaired performance in a T-maze was seen in rats exposed to restraint stress prior to testing [48], but not after 4 hours of recovery from restraint stress [49], suggesting that delay-dependent attention deficits rather than working memory disruption may explain these performance problems. Apart from direct effects on attention, however, both chronic and acute stress have been discussed to impair spatial memory and learning capacities in rodents [50]–[57]. Interestingly, a recent study on stress-induced impairment of a delayed-response task found that stress is particularly detrimental to the ability to update and maintain information throughout a delay period [58].

In the present study, stress sensitivity and, hence, memory performance seem to be most affected in cLH rats tested with short retention interval. Similarly, anhedonic-like behaviour could only be triggered by electric foot-shock stress in cLH rats [12], assuming that some depressive-like symptoms seem to require an additional external stressor to manifest. Notably, exposure to an inescapable stressor has been proposed to increase attention or vigilance to sensory stimuli and, hence, to the response to those stimuli [59]. The difference between cLH and cNLH rats in responding to the stressful procedure may therefore be explained by a difference in attention or vigilance to external cues [60]. Overall, the cLH rats’ cognitive impairments that occurred in combination with the stressful procedure are similar to the deficits of attention and working memory observed in patients with major depression [3], [4], [61], [62]. The assumption that these impairments may be caused by enhanced stress sensitivity opens up an intriguing possibility: Translating back this hypothesis to human major depression and considering stress effects systematically in the study and analysis of major depression could help to explain the variability of results often seen in clinical populations.

### Where have I been? Where Should I go?

The DSWS task is particularly relevant for assessing PFC and HC deficits as it is a complex decision making task that relies on both working and intermediate memory as well as on memory-guided decision making. To solve the DSWS task, the rat relies on both retrospective and prospective working memory. The subject may thus encode either already visited arms (retrospective; Where have I been?) or still-to-be-visited arms (prospective; Where should I go?). In a series of experiments it has been shown that rats seem to use a flexible, dual-code memory system, being able to translate back and forth between (presumably more HC-dependent) retrospective and (presumably more PFC-dependent) prospective codes [19], [21]. Variations in the length of the delay differentially seem to favour prospective and retrospective coding, with longer delays (60 min) evoking retrospective encoding [19], and shorter delays (15 min) allowing for both pro- and retrospective codes [19], [21]. Although rats were tested with two different delay durations in the present study, we observed a general recency effect (i.e. rats were more likely to avoid revisiting more recent choices than choices made earlier), implying the use of retrospective codes in both strains and delay conditions. Future studies may include longer delays or various training arm subsets to bear novel insights on how different memory systems are adaptively utilized and interact during decision making.

### Individual Differences and the Nature of Learning

To describe the learning progress on the radial arm maze, sigmoid-function-like performance gains have been suggested [63]–[65]. Fitting sigmoid functions to each individual’s performance scores, we observed substantial variation between subjects in baseline performance (the level already obtained on the first 2 days), learning amplitude, learning speed (captured by the slope of the sigmoid), and training day at which the maximum gain in learning occurred (centre point of the sigmoid). Beyond the effects of genotype and/or environment on the individual’s behaviour, personality-like features have also been discussed in this context to shape an animal’s unique behavioural phenotype [66], [67]. Interestingly, many learning curves showed an abrupt, almost step-like increase from a baseline level of responding to a stable level. In line with previous studies [38], [64], this abrupt change in behaviour typically occurred within the first few trials. In other examples, rather slow and smoothly changing improvements were observed, suggesting that some animals may rely on a more gradual learning system or apply a more cautious choice criterion. Furthermore, the post-acquisition level was characterized by still quite large fluctuations in most animals, putting into question the existence of an asymptote in the strict sense [64]. In fact, the longer plateau performance could be assessed, the larger the variations in post-acquisition performance seemed to be. This kind of variability may either reflect noise in repeated measurements [68], or may be explained through more systematic behavioural effects due to overtraining (e.g. [69], [70]). A motivational deficit may also explain why there was one animal that appeared to fall off in memory performance over the course of testing (**Figure 5E**). Finally, there was one cLH rat in the 30 s condition characterized by a negative plateau value, indicating that this animal may not have learned the task.

### Conclusions

With our study of higher executive functions in the rat learned helplessness model, we provide direct animal homologues of clinically important measures in human research, and contribute to the non-invasive assessment of cognitive impairments associated with depression. Specifically, we could show that memory performance was most impaired in cLH rats tested with a short delay, suggesting a stress-related disruption of attentional processes in animals that are sensitive to stress. Because similar attention and working memory deficits have been observed in patients with major depression, future studies may help to shed light on the role of stress as a critical causal and maintenance factor of cognitive deficits.
